# Efficient trapping of HIV-1 envelope protein by hetero-oligomerization with an N-helix chimera

**DOI:** 10.1186/1742-4690-2-51

**Published:** 2005-08-10

**Authors:** Wu Ou, Jonathan Silver

**Affiliations:** 1 Laboratory of Molecular Microbiology, National Institute of Allergy and Infectious Diseases, National Institutes of Health, Building 4, Room 336, Bethesda, MD 20892, USA

## Abstract

**Background:**

The N-heptad repeat region of the HIV-1 Transmembrane Envelope protein is a trimerization domain that forms part of a "six helix bundle" crucial to Envelope-mediated membrane fusion. N-heptad repeat peptides have been used as extracellular reagents to inhibit virus fusion.

**Results:**

When expressed intracellularly with wild-type HIV-1 Envelope protein, the N-heptad repeat domain efficiently hetero-oligomerized with Envelope and trapped it in the endoplasmic reticulum or early Golgi, as indicated by lack of transport to the cell surface, absent proteolytic processing, and aberrant glycosylation.

**Conclusion:**

Post-translational processing of HIV Envelope is very sensitive to an agent that binds to the N-heptad repeat during synthesis, suggesting that it might be possible to modify drugs that bind to this region to have transport-blocking properties.

## Background

Retroviral envelope proteins (Env) are synthesized as precursor proteins in the secretory pathway. After co-translational transfer to the endoplasmic reticulum (ER), the envelope precursor trimerizes and becomes extensively glycosylated. On passage through the medial- and trans-Golgi, sugar residues are trimmed and modified, and Env is proteolytically cleaved by a furin-like enzyme into Surface (SU) and Transmembrane (TM) moieties [1-6]. Trimerization is largely determined by a ~ 30 amino acid alpha-helical domain near the amino-terminus of TM designated the N-heptad repeat or N-helix, residues on one side of which associate hydrophobically to form a trimeric "coiled coil" [7-10]. In the case of HIV and related lentiviruses, about 50 amino acids downstream of the N-heptad repeat is another domain that forms an alpha-helix during rearrangements associated with receptor-binding and membrane fusion. This C-helix region of each Env monomer folds back and binds in an anti-parallel orientation in grooves between N-helix monomers to form a thermodynamically stable, "6-helix bundle" whose structure has been determined [8-10]. Formation of the 6-helix bundle is thought to drive fusion by pulling virus and target cell membranes together [11-16]. Subtle interactions between helix residues that do not affect 6-helix bundle thermal stability also impact fusion [17].

Because of their structural and mechanistic importance for fusion, the N and C-helix regions are targets for therapeutic peptides and drugs. C-helix peptides inhibit fusion at nanomolar concentration [18-20]. Extensive structural and mutagenesis studies have shown that they work, at least in part, by competing with the C-helix for binding to the N-helix trimer [21-25]. Three bulky hydrophobic side chains at one end of the C-helix fit into a deep hydrophobic pocket in the N-helix trimer that has been proposed as a target for small molecule drugs[22]. N-helix peptides are less potent fusion inhibitors, requiring micromolar concentration[26]. Two mechanisms have been proposed for their action: forming homotrimers that bind viral C-helices, and forming heterotrimers with viral N-helix monomers[27]. When N-helix peptides are added extracellularly, forming heterotrimers requires peptide exchange with monomers in pre-formed virus trimer, which may be inefficient.

We previously reported that when Moloney-murine leukemia virus (Mo-MLV) N-helix was expressed intracellularly as a chimeric protein, it formed heterotrimers with co-expressed wild-type Mo-MLV Env, which blocked transport to the cell surface[28]. The heterotrimers were apparently trapped in the ER since Env in the heterotrimer had an immature glycosylation pattern and was not cleaved into SU and TM, although it could be cleaved by furin *in vitro*[28]. We now show that similar trapping of HIV-1 Env occurs in cells expressing an HIV-1 N-helix-YFP chimeric protein. The trapping is remarkably efficient as no proteolytically cleaved, heterotrimeric molecules were detectable by Western blot, implying that heterotrimeric molecules do not reach the late Golgi. The strength of the trapping suggests that small molecule drugs that bind N-helix in the ER might be engineered to block subsequent trafficking and thereby inhibit assembly of infectious particles.

## Results and Discussion

**Figure 1:**
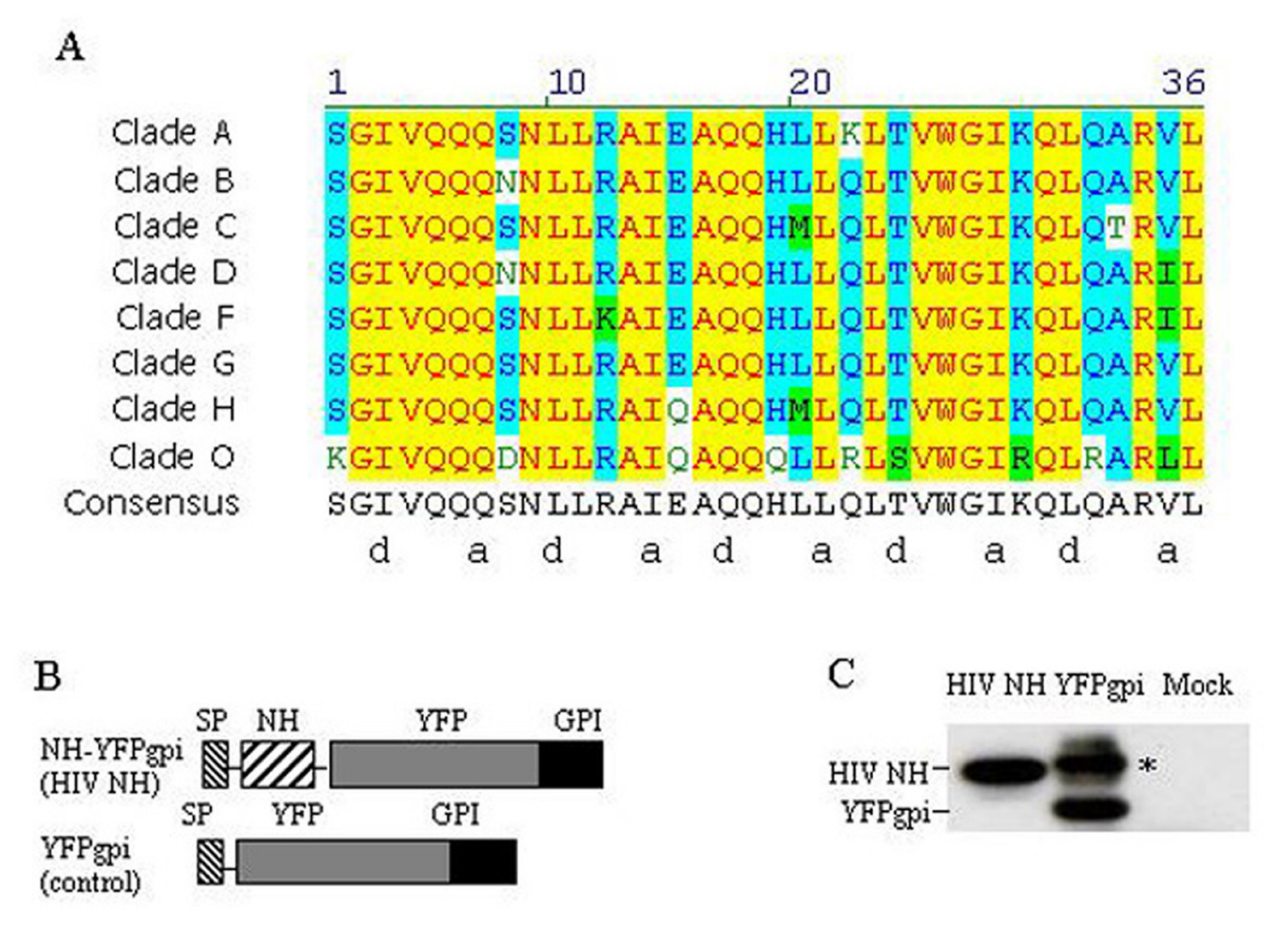
A. Comparison of consensus amino acid sequences of N-helix regions from various HIV-1 clades, and the consensus sequence that was used to make pNH-YFPgpi. The letters a and d under the consensus sequence indicate the position of corresponding amino acids on a helical wheel. B. Schematic diagram of the coding sequence regions in expression plasmids pYFPgpi and pNH-YFPgpi. SP, signal peptide; YFP, yellow fluorescent protein; NH, N-helix; GPI, gpi attachment signal. C. Western blot with anti-GFP antiserum of HeLa cells transfected with pNH-YFPgpi (lane 1), pYFPgpi (lane 2), or untransfected HeLa cells (lane 3). *, aberrant YFPgpi product. One of three independent experiments with similar results is shown.

The amino acid sequence of the HIV N-helix is remarkably conserved among isolates, especially in the helical wheel "a" and "d" positions that mediate trimer association (Figure 1A). We chose a consensus sequence for the N-helix and inserted it in frame between a signal sequence and the yellow fluorescent protein (YFP) gene in a CMV promoter-driven, cell-surface expression vector with a glycosylphosphatidylinositol (gpi) membrane linkage sequence (pYFPgpi)[29] to make pNH-YFPgpi (Figure 1B). We expected that the signal sequence in this construct would direct the nascent N-helix to the secretory pathway where it could interact with co-expressed HIV Envelope, and the YPF provided a convenient tag for visualization and immunoprecipitation (see below). HEK293 cells transfected with this plasmid expressed YFP mainly on the cell surface in a pattern indistinguishable from that induced by pYFPgpi[28] (data not shown). Western blot analysis using anti-GFP antibody showed that HEK293 cells transfected with the N-helix expression plasmid contained the expected 40 kD YFP fusion protein, versus a 36 kD YFP product in cells transfected with the parent vector lacking the N-helix insertion (Figure 1C). As noted previously[28], the parent vector, pYFPgpi, also generated a higher molecular weight YFP species possibly due to aberrant glycosylation (* in figure 1C, lane 2).

**Figure 2:**
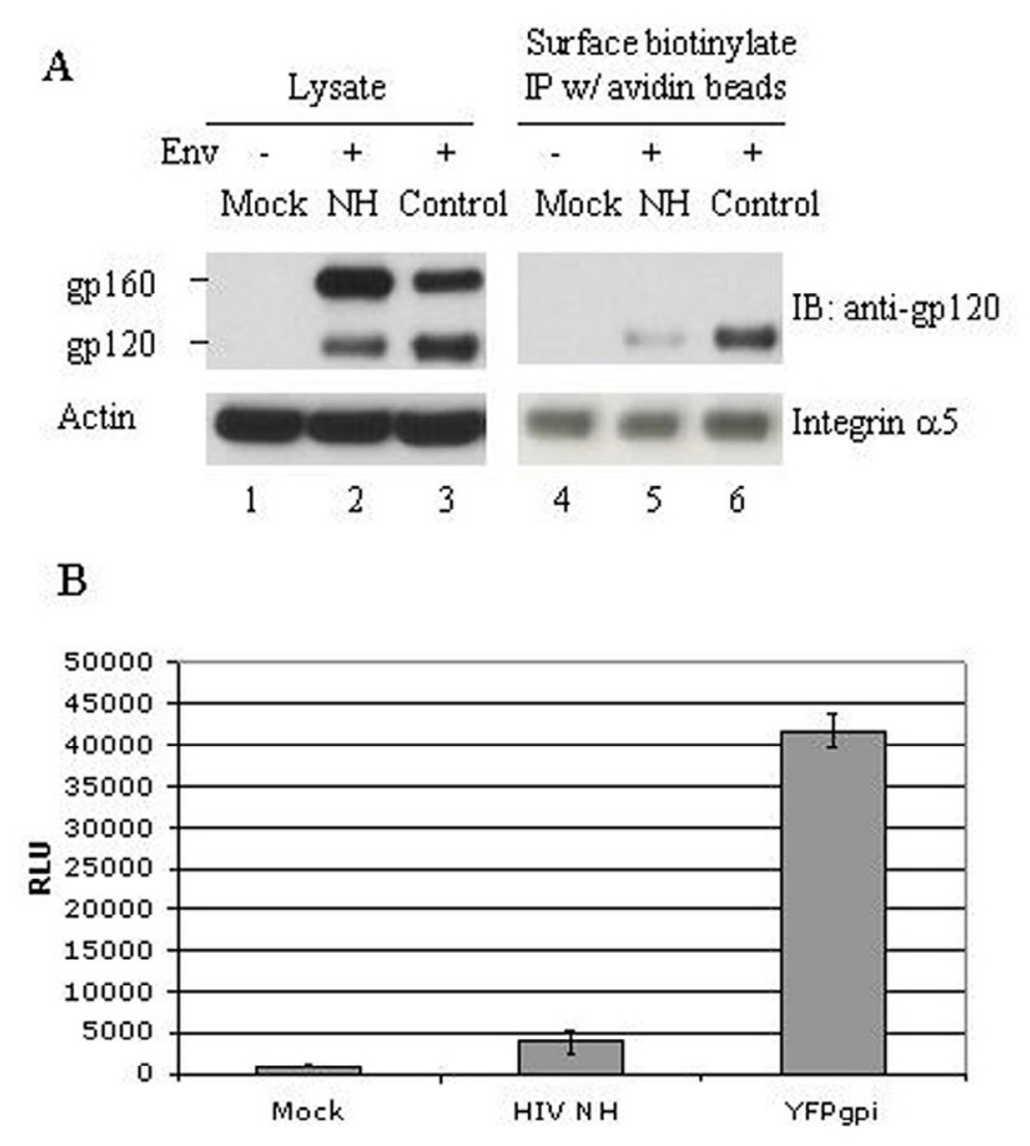
A. Western blot analysis of HeLa cells untransfected (lanes 1, 4), or transfected with an expression vector for HIV-1 Env and Tat plus either pNH-YFPgpi (lanes 2, 5) or pYFPgpi as control (lanes 3, 6). Left side, cell lysates analyzed with rabbit anti-gp120 antiserum (upper panel), or anti-actin as loading control (lower panel). Right side, cell surface proteins labeled with NHS-biotin, precipitated with avidin-agarose, and analyzed with rabbit anti-gp120 antiserum (upper panel), or anti-integrin α5 as loading control (lower panel). One of two independent experiments with similar results is shown. B. Cell fusion assay. Indicator HeLa-TZM cells (CD4+, CXCR4+, CCR5+, containing an HIV LTR-luciferase reporter) were cultured overnight with HEK293 cells untransfected (left bar) or transfected with an expression vector for pNL4-3 strain HIV-1 Env and Tat, plus either pNH-YFPgpi (middle bar) or pYFPgpi (right bar) and analyzed for luciferase activity. RLU, relative light units. One of four independent experiments with similar results is shown.

To see if the N-helix-YFP fusion protein affected synthesis or trafficking of wild-type HIV-1 Env, we co-transfected HeLa cells with an expression vector for HIV-1 Env strain AD8 (pAD8) plus either pNH-YFPgpi or pYFPgpi as a control. Western blot analysis of whole cell lysates using polyclonal anti-gp120 (SU) antiserum showed that the N-helix fusion protein partially inhibited processing the gp160 Env precursor to gp120 (Figure 2A, lane 2 versus lane 3). The total amount of Env protein was not affected. Western blot with anti-actin antibody showed that equal amounts of protein were loaded in all samples (Figure 2A, left lower panel).

The partial inhibition of Env processing was associated with a more striking inhibition of transport to the cell surface, evaluated by biotinylating cell-surface proteins with biotin-NHS, precipitating biotinylated proteins with avidin-agarose, and analyzing the precipitated proteins by Western blot using anti-gp120 antiserum. Co-expressed N-helix fusion protein markedly reduced cell surface gp-120 (Figure 2A, lane 5 versus lane 6). Western blot using antibody to integrin alpha5 showed that equal amounts of biotinylated cell surface proteins were loaded in all lanes (Figure 2A, right lower panel). The absence of a biotinylated form of gp160 shows that the biotin label did not attach to intracellular proteins.

The reduction in cell surface gp120 was associated with a comparable reduction in cell fusion activity, measured using a standard assay in which HeLa cells or HEK293 cells transfected with plasmids which express HIV-1 Tat as well as Env were mixed with indicator HeLa-TZM cells that express HIV receptor (CD4) and co-receptors (CXCR4 and CCR5), and contain a luciferase reporter driven by the HIV-1 LTR. Cell fusion induced by a CXCR4-tropic Env (derived from pNL4-3) was reduced 8- to 10-fold by co-expression of the N-helix fusion protein, compared to co-expression of the control YFPgpi (Figure 2B, lower panel). Cell fusion induced by a CCR5-tropic Env (derived from pAD8) was reduced 2–5 fold in 3 comparable experiments. Lower inhibition in the case of the CCR5-tropic Env may be due to greater expression of Env by the pAD8Env vector compared to the pNL4-3Env vector (unpublished observations), and/or to greater expression of CCR5 than CXCR4 by the TZM indicator cells, which were engineered to overexpress CCR5.

**Figure 3:**
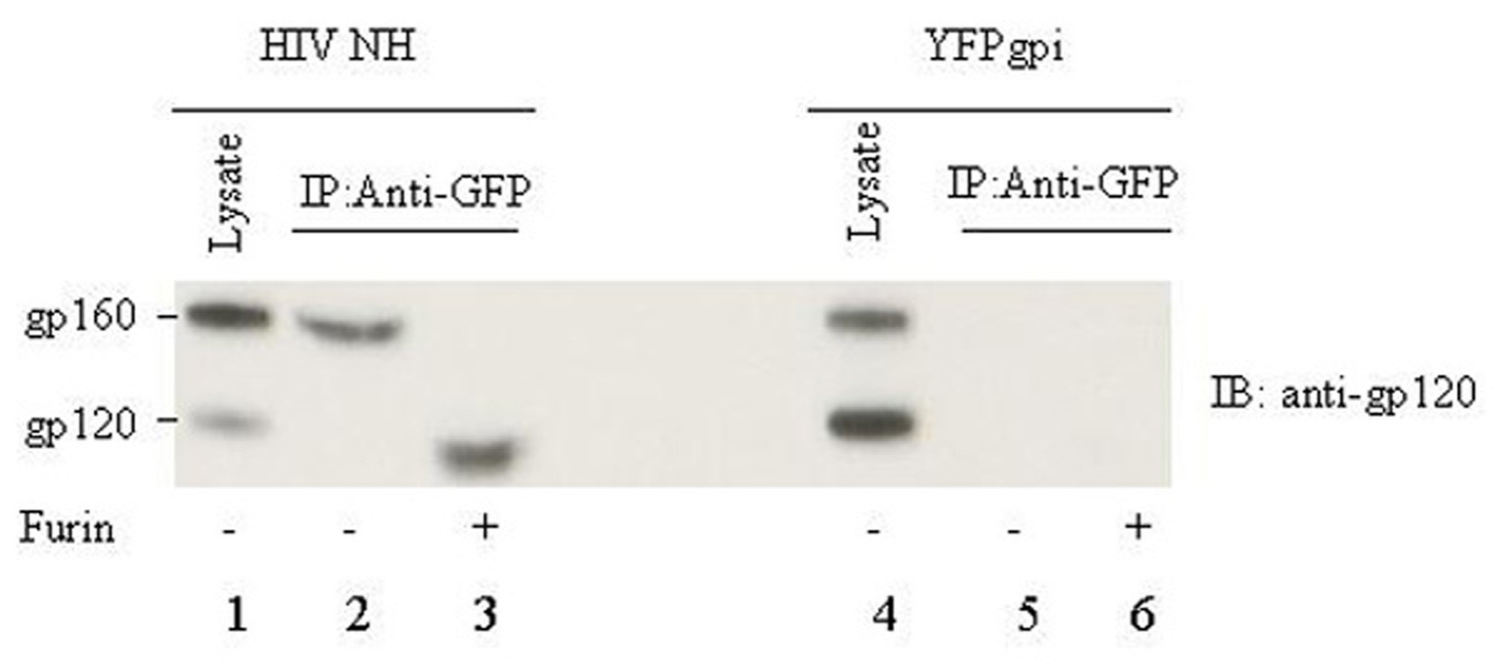
Western blot analysis of HeLa cells transfected with an expression vector for HIV-1 Env plus either pNH-YFPgpi (lanes 1–3) or pYFPgpi as control (lanes 4–6). Total cell lysates (lanes 1, 4) or anti-GFP immunoprecipitates (lanes 2, 3, 5, 6) were treated with furin (lanes 3, 6) or mock treated (lanes 1, 2, 4, 5) and analyzed with rabbit anti-gp120 antiserum.

To see if the N-helix-YFP fusion protein physically associated with HIV-1 Env, we immuno-precipitated cell lysates with anti-GFP antibody and analyzed the immunoprecipitates by Western blot using anti-gp120 antiserum. In cells co-transfected with pNH-YFPgpi plus the HIV-1 Env expression vector, anti-GFP antibody co-immunoprecipitated the Env precursor gp160 but not gp-120, even though gp120 was present in the cell lysate (Figure 3, lane 2 versus lane 1). This shows that, to the limit of sensitivity of Western blot, all of the HIV Env that hetero-oligomerized with the N-helix fusion protein was prevented from being processed to gp120. A similar result was obtained in the case of MLV: the Env that co-immunoprecipitated with chimeric N-helix was not detectably proteolytically processed[28]. The small amount of Env that was processed to SU in the current experiments (Figure 3, lane 1) presumably came from wild-type Env molecules that homotrimerized rather than forming hetero-oligomers with N-helix-YFP. In control cells co-transfected with pYFPgpi instead of pNH-YFPgpi, the Env precursor was more efficiently processed to gp120, as expected (Figure 3, lane 4 versus lane 1), and the anti-GFP antibody did not co-immunoprecipitate HIV Env (lanes 4 and 5); the latter shows that the interaction between N-helix-YFP and Env was not due to non-specific stickiness of YFP.

To see if the processing defect of the Env precursor complexed with N-helix-YFP was due to resistance of this form of Env to furin, we incubated the immunoprecipitates with furin enzyme. Exogenous furin cleaved the co-immunoprecipitated Env precursor to a species that migrated slightly faster than native gp120 (Figure 3, lane 3 versus lane 1). Altered mobility of the furin cleavage product is likely due to aberrant glycosylation. In similar experiments with MLV[28], the *in vitro *cleavage product of hetero-oligomerized Env treated with furin also migrated slightly faster than normal SU, but co-migrated with SU from cells treated with brefeldin A, a drug that disrupts the Golgi and blocks Golgi-associated sugar modifications[30]. Since the HIV Env precursor complexed with N-helix-YFP was cleavable *in vitro *but was not cleaved *in vivo*, the simplest interpretation of the data is that hetero-oligomerization of HIV Env gp160 with N-helix-YFP leads to arrest of this species in the ER or cis Golgi, preventing maturation of sugars and proteolytic cleavage that normally occur in the medial and trans Golgi. It is also possible that the hetero-oligomerized Env is misrouted to some other furin-negative compartment.

In comparable experiments with Mo-MLV we showed that blocking the ability of the MLV N-helix to trimerize by substituting proline for leucine in the center of the trimerization domain abolished its ability to trap Env in the ER, providing additional evidence that oligomerization was responsible for the trapping[28]. Further, the YFPgpi portion of the chimeric N-helix did not contribute to inhibition, since the MLV N-helix linked to a 9 amino acid HA epitope instead of YFPgpi was equally potent in trapping MLV Env in the ER[28]. Since neither YPF nor the HA epitope inhibit trafficking when attached to other proteins, we surmise that inclusion of N-helix by itself in a heterotrimer with Env causes misfolding.

Given the strong conservation of amino acids that direct N-helix trimerization, it is likely that intracellular expression of an N-helix chimera would inhibit processing of all strains of HIV. From a practical point of view, however, the dominant negative effect of N-helix constructs is limited by their level of expression in the ER compared to that of wild-type Env. Both the HIV and MLV N-helix-YFP fusion proteins are efficiently transported to the cell surface when expressed alone, based on the pattern of fluorescence in confocal microscopy, which is mainly restricted to the plasma membrane as previously shown[28]. In cells co-expressing Env, there was a slight increase in intracellular fluorescence but most of the fluorescence remained on the plasma membrane, suggesting that most N-helix-YFP molecules leave the ER before having a chance to hetero-oligomerize with Env. To attempt to block "premature" egress, which might reduce its ability to form a heterotrimer, we replaced the gpi attachment peptide signal with a "KDEL" ER retention signal to make pNH-YFP-KDEL. The KDEL construct was efficiently retained in the ER as judged by a reticular, cytoplasmic fluorescence pattern; however, it was not more inhibitory than the unmodified fusion protein when co-transfected with HIV Env in a cell fusion assay (data not shown). We also explored the effect of shortening the N-helix by deleting 7 or 14 amino acids (two or four alpha-helical turns) from either end, since short peptides can sometimes be induced to cross cell membranes by attaching a basic membrane transport domain[31]. However, the shorter N-helix versions were less inhibitory in the cell fusion assay than the full N-helix.

How do the N-helix chimeric proteins interact with HIV Env expressed in the secretory pathway? Like extracellular N-helix peptides, they could form heterotrimers with N-helix regions in Env molecules[32], or homotrimerize and then interact with C-helix regions in Env[33]. These possibilities might be distinguished by seeing how mutations in Env C-helix residues versus N-helix residues affect hetero-oligomerization with N-helix constructs. Extracellular N-helix peptides preferentially bind receptor-activated Env[33], presumably because the interacting N- or C-helix regions are poorly exposed in the mature, unactivated Env. Our observations imply that surfaces in Env that interact with N-helix chimeras are exposed in nascent Env. Our results do not exclude the possibility that N-helix-YFPgpi also inhibits fusion by interacting with receptor-activated Env on the cell surface.

An unexpected observation made in the course of these studies was that the control vector pYFPgpi inhibited fusion about ten-fold when transfected with HIV Env expression vectors. Therefore, to evaluate the effect of the N-helix we compared transfections with pNH-YFPgpi to transfections with pYFPgpi. The reason for inhibition by pYFPgpi is currently under investigation.

## Conclusion

The remarkable efficacy of trapping by hetero-oligomerization suggests a drug strategy of trying to engineer small molecules that bind the Env N-helix in the ER in a fashion that blocks trafficking. Small molecules that bind to the hydrophobic pocket at one end of the N-helix trimer are under development[22,34-37]. Coupling them to an ER retention signal like KDEL might inhibit Env trafficking. Macrocycle drugs such as cyclosporinA act as bivalent ligands that bring together two proteins, one of which can function as an ER chaperone (e.g., cyclophilinB)[38]. Structures of several of these macrocycle-chaperone complexes are known, and they show that only one side of the macrocycle is necessary for tight (nanomolar) binding to the chaperone[39,40]. Based on these results, it might be possible to engineer a bi-dentate drug, one portion of which binds in grooves of the HIV Env N-helix trimer while another portion binds an ER chaperone, promoting ER retention. A natural example related to this strategy was recently described: a small molecule intermediate in the cholesterol synthesis pathway (farnesol) that binds an ER-associated enzyme in this pathway (HMG-CoA reductase), resulting in accelerated degradation of the enzyme[41]. The idea we propose is the "flip side" of a hunt for small molecules that inhibit protein misfolding[42,43]. HIV Env may provide a propitious target for drug-induced trapping since it is naturally inefficiently processed[4] and HIV virions from several strains bear very few Env trimers on their surface[44,45].

## Materials and methods

### Constructs

We aligned N-helix amino acid sequences of HIV-1 envelopes in the Los Alamos database  and generated a consensus sequence for each clade (A, B, C, D, F, G, H and O), then generated the consensus sequence for all the clades (Fig. 1A), which is the N-helix sequence used in this paper. Oligonucleotides encoding this HIV-1 N-helix with Sal I restriction enzyme overhanging sequences were synthesized, annealed and ligated into plasmid pYFP-gpi[29] at the Sal I site, to generate plasmid pNH-YFPgpi (Figure 1B). The oligonucleotide sequences used were: 5' tcgacttctggtatagtgcagcagcagaacaatttgctgagggctattgaggcgcaacagcatctgtt-gcaactcacagtctggggcatcaaacagctccaggcaagagtcctggcg 3', and 5' tcgacgccaggactcttgcct-ggagctgtttgatgccccagactgtgagttgcaacagatgctgttgcgcctcaatagccctcagcaaattgttctgctgctgcactataccagaag 3'. For expression of T-tropic (CXCR4-using) and M-tropic (CCR5-using) HIV-1 Env, we used plasmids pdl1443 and pAD8Env, respectively, which were derived from molecular clones pNL4-3 and pAD8 by deleting 3.1 kb of gag sequences between SphI and MscI sites [46]. These plasmids express HIV-1 Tat as well as Env.

### Transfection, surface protein labeling and cell fusion

HEK 293 or HeLa cells were co-transfected with Env-expressing constructs pdl1443 or pAD8Env, plus pNH-YFPgpi or pYFP-gpi as control, using Lipofectamine2000 (Invitrogen, Carlsbad, CA). Twenty four to 48 hours later, the cells were rinsed with phosphate buffered saline (PBS) and labeled on ice with 1 mg/ml Sulfo-NHS-LC-LC biotin (Pierce, Rockford, IL) in PBS for one hour. After labeling, the biotinylation reagent was quenched with 100 mM glycine in PBS buffer. Following PBS wash, some of the cells were lysed with RIPA lysis buffer (150 mM NaCl, 1% Triton X-100, 0.1% SDS, 0.5% sodium deoxycholate) for immunoprecipitation or direct western blot, and the remainder of the cells were co-cultivated with TZM-bl cells[47,48] overnight and then assayed for luciferase activity (Promega, Madison, WI) as described[28].

### Immunoprecipitation, furin cleavage *in vitro *and western blot

To immunoprecipitate cell surface biotin-labeled HIV-1 Env protein, avidin beads were directly added to the labeled cell lysate. After binding for 2 hours with agitation, beads were washed with lysis buffer and PBS, and bound proteins eluted by boiling for 3 min in 1X SDS-PAGE sample buffer (Invitrogen). The eluate was run on a 4–12% SDS-PAGE, transferred to PVDF membrane, and blotted with polyclonal anti-gp120 serum (a gift from Klaus Strebel, LMM/NIAID, made by immunizing rabbits with purified gp120 of HIV-1 strain IIIB), or with anti-integrin α5 (BD Transduction Lab, San Diego, CA) as a control. To cross-immunoprecipitate intracellular HIV-1 Env protein, cell lysates were pre-cleared with normal mouse serum and protein G Sepharose beads (Amersham, Piscataway, NJ) for 4 hour at 4°C with agitation. The supernatant was collected and immunoprecipitated with monoclonal anti-GFP antibody (Clontech, Palo Alto, CA) overnight and protein G beads for an additional 2 hours. Beads were washed 3 times with lysis buffer and 2 times with PBS buffer. Protein was eluted from one half of the beads by boiling in 1X SDS-PAGE sample buffer, and the remaining beads were re-suspended in furin reaction buffer (0.5% triton X-100, 1 mM CaCl_2_, 100 mM HEPES, 1 mM β-mercaptoethanol) and treated with 0.578 mg/ml furin (R&D systems, Minneapolis, MN) at 37°C for 16 hours as descibed[28]. The reaction was stopped and protein eluted by boiling in 1× SDS-PAGE sample buffer. The eluted protein was analyzed by western blot using rabbit anti-gp120 serum.

## Competing interests

The author(s) declare that they have no competing interests.

## Authors' contributions

WO carried out the studies, participated in the design and conception of the project, and helped draft the manuscript. JS participated in the design and conception of the project and drafted the manuscript. Both authors read and approved the final manuscript.

## References

[B1] Dewar RL, Natarajan V, Vasudevachari MB, Salzman NP (1989). Synthesis and processing of human immunodeficiency virus type 1 envelope proteins encoded by a recombinant human adenovirus. J Virol.

[B2] Doms RW, Earl PL, Moss B (1991). The assembly of the HIV-1 env glycoprotein into dimers and tetramers. Adv Exp Med Biol.

[B3] Earl PL, Moss B, Doms RW (1991). Folding, interaction with GRP78-BiP, assembly, and transport of the human immunodeficiency virus type 1 envelope protein. J Virol.

[B4] Willey RL, Bonifacino JS, Potts BJ, Martin MA, Klausner RD (1988). Biosynthesis, cleavage, and degradation of the human immunodeficiency virus 1 envelope glycoprotein gp160. Proc Natl Acad Sci U S A.

[B5] Hallenberger S, Moulard M, Sordel M, Klenk HD, Garten W (1997). The role of eukaryotic subtilisin-like endoproteases for the activation of human immunodeficiency virus glycoproteins in natural host cells. J Virol.

[B6] Merkle RK, Helland DE, Welles JL, Shilatifard A, Haseltine WA, Cummings RD (1991). gp160 of HIV-I synthesized by persistently infected Molt-3 cells is terminally glycosylated: evidence that cleavage of gp160 occurs subsequent to oligosaccharide processing. Arch Biochem Biophys.

[B7] Center RJ, Lebowitz J, Leapman RD, Moss B (2004). Promoting trimerization of soluble human immunodeficiency virus type 1 (HIV-1) Env through the use of HIV-1/simian immunodeficiency virus chimeras. J Virol.

[B8] Chan DC, Fass D, Berger JM, Kim PS (1997). Core structure of gp41 from the HIV envelope glycoprotein. Cell.

[B9] Weissenhorn W, Dessen A, Harrison SC, Skehel JJ, Wiley DC (1997). Atomic structure of the ectodomain from HIV-1 gp41. Nature.

[B10] Tan K, Liu J, Wang J, Shen S, Lu M (1997). Atomic structure of a thermostable subdomain of HIV-1 gp41. Proc Natl Acad Sci U S A.

[B11] Lu M, Stoller MO, Wang S, Liu J, Fagan MB, Nunberg JH (2001). Structural and functional analysis of interhelical interactions in the human immunodeficiency virus type 1 gp41 envelope glycoprotein by alanine-scanning mutagenesis. J Virol.

[B12] Weissenhorn W, Dessen A, Calder LJ, Harrison SC, Skehel JJ, Wiley DC (1999). Structural basis for membrane fusion by enveloped viruses. Mol Membr Biol.

[B13] Melikyan GB, Markosyan RM, Hemmati H, Delmedico MK, Lambert DM, Cohen FS (2000). Evidence that the transition of HIV-1 gp41 into a six-helix bundle, not the bundle configuration, induces membrane fusion. J Cell Biol.

[B14] Cao J, Bergeron L, Helseth E, Thali M, Repke H, Sodroski J (1993). Effects of amino acid changes in the extracellular domain of the human immunodeficiency virus type 1 gp41 envelope glycoprotein. J Virol.

[B15] Ji H, Shu W, Burling FT, Jiang S, Lu M (1999). Inhibition of human immunodeficiency virus type 1 infectivity by the gp41 core: role of a conserved hydrophobic cavity in membrane fusion. J Virol.

[B16] Mo H, Konstantinidis AK, Stewart KD, Dekhtyar T, Ng T, Swift K, Matayoshi ED, Kati W, Kohlbrenner W, Molla A (2004). Conserved residues in the coiled-coil pocket of human immunodeficiency virus type 1 gp41 are essential for viral replication and interhelical interaction. Virology.

[B17] Suntoke TR, Chan DC (2005). The fusion activity of HIV-1 gp41 depends on interhelical interactions. J Biol Chem.

[B18] Jiang S, Lin K, Strick N, Neurath AR (1993). HIV-1 inhibition by a peptide. Nature.

[B19] Eckert DM, Kim PS (2001). Mechanisms of viral membrane fusion and its inhibition. Annu Rev Biochem.

[B20] Wild C, Greenwell T, Matthews T (1993). A synthetic peptide from HIV-1 gp41 is a potent inhibitor of virus-mediated cell-cell fusion. AIDS Res Hum Retroviruses.

[B21] Lu M, Blacklow SC, Kim PS (1995). A trimeric structural domain of the HIV-1 transmembrane glycoprotein. Nat Struct Biol.

[B22] Chan DC, Chutkowski CT, Kim PS (1998). Evidence that a prominent cavity in the coiled coil of HIV type 1 gp41 is an attractive drug target. Proc Natl Acad Sci U S A.

[B23] Wild C, Greenwell T, Shugars D, Rimsky-Clarke L, Matthews T (1995). The inhibitory activity of an HIV type 1 peptide correlates with its ability to interact with a leucine zipper structure. AIDS Res Hum Retroviruses.

[B24] Rimsky LT, Shugars DC, Matthews TJ (1998). Determinants of human immunodeficiency virus type 1 resistance to gp41-derived inhibitory peptides. J Virol.

[B25] Furuta RA, Wild CT, Weng Y, Weiss CD (1998). Capture of an early fusion-active conformation of HIV-1 gp41. Nat Struct Biol.

[B26] Wild C, Oas T, McDanal C, Bolognesi D, Matthews T (1992). A synthetic peptide inhibitor of human immunodeficiency virus replication: correlation between solution structure and viral inhibition. Proc Natl Acad Sci U S A.

[B27] Bewley CA, Louis JM, Ghirlando R, Clore GM (2002). Design of a novel peptide inhibitor of HIV fusion that disrupts the internal trimeric coiled-coil of gp41. J Biol Chem.

[B28] Ou W, Silver J (2005). Inhibition of murine leukemia virus envelope protein (env) processing by intracellular expression of the env N-terminal heptad repeat region. J Virol.

[B29] Keller P, Toomre D, Diaz E, White J, Simons K (2001). Multicolour imaging of post-Golgi sorting and trafficking in live cells. Nat Cell Biol.

[B30] Misumi Y, Misumi Y, Miki K, Takatsuki A, Tamura G, Ikehara Y (1986). Novel blockade by brefeldin A of intracellular transport of secretory proteins in cultured rat hepatocytes. J Biol Chem.

[B31] Wadia JS, Dowdy SF (2005). Transmembrane delivery of protein and peptide drugs by TAT-mediated transduction in the treatment of cancer. Adv Drug Deliv Rev.

[B32] Caffrey M, Kaufman J, Stahl S, Wingfield P, Gronenborn AM, Clore GM (1999). Monomer-trimer equilibrium of the ectodomain of SIV gp41: insight into the mechanism of peptide inhibition of HIV infection. Protein Sci.

[B33] He Y, Vassell R, Zaitseva M, Nguyen N, Yang Z, Weng Y, Weiss CD (2003). Peptides trap the human immunodeficiency virus type 1 envelope glycoprotein fusion intermediate at two sites. J Virol.

[B34] Eckert DM, Malashkevich VN, Hong LH, Carr PA, Kim PS (1999). Inhibiting HIV-1 entry: discovery of D-peptide inhibitors that target the gp41 coiled-coil pocket. Cell.

[B35] Ferrer M, Kapoor TM, Strassmaier T, Weissenhorn W, Skehel JJ, Oprian D, Schreiber SL, Wiley DC, Harrison SC (1999). Selection of gp41-mediated HIV-1 cell entry inhibitors from biased combinatorial libraries of non-natural binding elements. Nat Struct Biol.

[B36] Zhou G, Ferrer M, Chopra R, Kapoor TM, Strassmaier T, Weissenhorn W, Skehel JJ, Oprian D, Schreiber SL, Harrison SC, Wiley DC (2000). The structure of an HIV-1 specific cell entry inhibitor in complex with the HIV-1 gp41 trimeric core. Bioorg Med Chem.

[B37] Debnath AK, Radigan L, Jiang S (1999). Structure-based identification of small molecule antiviral compounds targeted to the gp41 core structure of the human immunodeficiency virus type 1. J Med Chem.

[B38] Mikol V, Kallen J, Walkinshaw MD (1994). X-ray structure of a cyclophilin B/cyclosporin complex: comparison with cyclophilin A and delineation of its calcineurin-binding domain. Proc Natl Acad Sci U S A.

[B39] Sedrani R, Kallen J, Martin Cabrejas LM, Papageorgiou CD, Senia F, Rohrbach S, Wagner D, Thai B, Jutzi Eme AM, France J, Oberer L, Rihs G, Zenke G, Wagner J (2003). Sanglifehrin-cyclophilin interaction: degradation work, synthetic macrocyclic analogues, X-ray crystal structure, and binding data. J Am Chem Soc.

[B40] Kallen J, Sedrani R, Zenke G, Wagner J (2005). Structure of human cyclophilin a in complex with the novel immunosuppressant sanglifehrin a at 1.6 a resolution. J Biol Chem.

[B41] Shearer AG, Hampton RY (2005). Lipid-mediated, reversible misfolding of a sterol-sensing domain protein. EMBO J.

[B42] Gestwicki JE, Crabtree GR, Graef IA (2004). Harnessing chaperones to generate small-molecule inhibitors of amyloid beta aggregation. Science.

[B43] Tanaka M, Machida Y, Nukina N (2005). A novel therapeutic strategy for polyglutamine diseases by stabilizing aggregation-prone proteins with small molecules. J Mol Med.

[B44] Chertova E, Bess JJWJ, Crise BJ, Sowder II RC, Schaden TM, Hilburn JM, Hoxie JA, Benveniste RE, Lifson JD, Henderson LE, Arthur LO (2002). Envelope glycoprotein incorporation, not shedding of surface envelope glycoprotein (gp120/SU), Is the primary determinant of SU content of purified human immunodeficiency virus type 1 and simian immunodeficiency virus. J Virol.

[B45] Zhu P, Chertova E, Bess JJ, Lifson JD, Arthur LO, Liu J, Taylor KA, Roux KH (2003). Electron tomography analysis of envelope glycoprotein trimers on HIV and simian immunodeficiency virus virions. Proc Natl Acad Sci U S A.

[B46] Felser JM, Klimkait T, Silver J (1989). A syncytia assay for human immunodeficiency virus type I (HIV-I) envelope protein and its use in studying HIV-I mutations. Virology.

[B47] Derdeyn CA, Decker JM, Sfakianos JN, Wu X, O'Brien WA, Ratner L, Kappes JC, Shaw GM, Hunter E (2000). Sensitivity of human immunodeficiency virus type 1 to the fusion inhibitor T-20 is modulated by coreceptor specificity defined by the V3 loop of gp120. J Virol.

[B48] Wei X, Decker JM, Liu H, Zhang Z, Arani RB, Kilby JM, Saag MS, Wu X, Shaw GM, Kappes JC (2002). Emergence of resistant human immunodeficiency virus type 1 in patients receiving fusion inhibitor (T-20) monotherapy. Antimicrob Agents Chemother.

